# SAW Humidity Sensor Sensitivity Enhancement via Electrospraying of Silver Nanowires

**DOI:** 10.3390/s16122024

**Published:** 2016-11-30

**Authors:** Farid Sayar Irani, Bahadir Tunaboylu

**Affiliations:** 1 Istanbul Sehir University, Department of Industrial Engineering, Altunizade Mh., Kusbakisi Cad., No. 27, Uskudar-Istanbul 34662, Turkey; bahadir.tunaboylu@tubitak.gov.tr; 2 Tubitak Marmara Research Center, PK 21, Gebze, Kocaeli 41470, Turkey

**Keywords:** humidity sensor, silver nanowire, surface acoustic wave (SAW) sensor, electrospray

## Abstract

In this research, we investigated the influence of the surface coatings of silver nanowires on the sensitivity of surface acoustic wave (SAW) humidity sensors. Silver nanowires, with poly(vinylpyrrolidone) (PVP), which is a hydrophilic capping agent, were chemically synthesized, with an average length of 15 µm and an average diameter of 60 nm. Humidity sensors, with 433 MHz frequency dual-port resonator Rayleigh-SAW devices, were coated by silver nanowires (AgNWs) using the electrospray coating method. It was demonstrated that increasing thickness of coated AgNW on the surfaces of SAW devices results in increased sensitivity. The highest frequency shift (262 kHz) in these SAW devices was obtained with an injection of 0.5 mL of the AgNW solution with a concentration of 0.5 mg/mL at an injection rate of 1 mL/h. It also showed the highest humidity sensitivity among the other prepared SAW devices.

## 1. Introduction

The sensing and sensors play an increasingly critical role in various industries. Humidity is a very important and common component in our environment, its measurement and control is not only vital for human comfort and health but also for different industries and technologies [1]. Different units are used for the measurement of vapor, and the most common ones are relative humidity (RH), dew frost point (D/FPT), and parts per million (PPM) [2].

Humidity sensors have gained increasing application in industrial processing and environmental control, such as in agriculture, where they are used for green house air conditioning, soil moisture, etc. Humidity control in manufacturing industries is crucial because it may affect the business cost of the product and the health and safety of personnel. Sensor sensitivity is a key factor in obtaining precise measurement results and as a consequence helps control the product costs.

Humidity sensors have been organized into three groups of optical sensors, electrical sensors, and acoustic sensors [3]. The relatively simple design and low price of the interrogation module are the two main advantages of electronic sensors [4]. However, they have several disadvantages, such as their response time, which is considered lengthy and varies from several tenths of a second to minutes. They need regular calibration and measurement, which would be difficult for relative humidity below 5%. Another drawback is the difficulty or sometimes impossibility of using them in certain critical environments, remote places, potentially explosive atmospheres, and areas with high electromagnetic interference. Optic sensors could be a viable alternative to electronic sensors in places using corrosive material. Moreover, because of their lack of electricity, there is no spark surrounding them, and it would be safe to monitor inflammable liquids or gases [4]. Acoustic methods of humidity measurements can be classified as mechanical methods. Existing methods of humidity measurement are based on surface acoustic waves (SAWs), the change in the resonance frequency of a quartz crystal microbalance (QCM), quartz tuning forks (QTF), film bulk acoustic resonator (FBAR) technology, and plate acoustic waves (Lamb waves). Recently, Lamb wave devices have also gained attention due to their high velocity, high quality factor (Q), and large coupling in comparison with SAW devices [5]. Acoustic sensors have several advantages over optic and electrical sensors such as a small power requirement, a low weight, and simple construction. Moreover, their working principle is based on frequency shift, which is deemed as a simple and accurate physical measurement [6].

The measurement of humidity by the acoustic method is based on the generation of a vibration that is affected by adsorption or absorption of water molecules on the sensing surface. The velocity and attenuation of waves in SAW sensors are changed by the adsorption of water molecules, which leads to a shift in frequency. The frequency shift can be detected by an oscillator with high accuracy. The SAW velocity can be affected by three basic interactions: a change in the film mass (mass-loading effect), a change in the conductivity of the film, and a change in the mechanical properties of the film material. From a practical point of view, for a gas-sensitive film, only two effects of the mass density of the film and the change in its electrical conductivity play an important role in sensor response [7]. On the other side it has been demonstrated that we can enhance the sensitivity of SAW sensors against various gases or volatile organic compounds with coating a different material on the surface of the sensor [8,9,10,11,12]. The mass loading *Δm* can be calculated as

(1) $$\Delta m=C_{s}V_{f}$$

where $C_{s}$ is the concentration of the solution of the sensitive material, and $V_{f}$ is the volume of the sensitive material. Due to mass loading, a frequency shift occurs, which can be calculated as follows:

(2) $$\Delta f=k^{2}f_{0}^{2}\frac{\Delta m}{A}$$

where *k* is the constant of the piezoelectric substrate, $f_{0}$ is the unperturbed resonant frequency of the SAW oscillator, and *A* is the sensitive film area [13,14].

In order to build a sensor with a high sensitivity for a certain gas, the chemical coating material should be chosen carefully to assure interaction between the target gas and the chemical coating material [15].

Humidity sensing materials can be grouped into two types: ceramics and polymers, which both have good chemical and thermal stability, have environmental adaptability, and can work in wide range of temperatures. Polymeric materials or porous ceramics have been widely used to enhance the performance of the humidity sensors, as they increase the capability of absorbing water molecules either through their specific material properties or their significantly increased surface areas [16]. A good sensitivity was achieved with humidity sensors coated with polymeric material were obtained in 2009 using silicon-containing polyelectrolyte [17].

Nanostructured material as a sensor-sensitive material has attracted great attention in recent years because their high surface-to-volume ratio provides highly active interfaces, which results in a sensitivity increase as well as a decrease in response and recovery times. Several nanomaterials have been used for preparing SAW humidity sensors, and good results have been obtained. Lei Sheng et al. [18] used a multi-walled carbon nanotube/Nafion (MWCNT/Nafion) composite material as a humidity-sensitive film that showed excellent sensitivity (above 400 kHz/% relative humidity (RH) in a range from 10% RH to 80% RH) and a short response time (~3 s@63%). 

Silver and its nanostructures are utilized as different sensors, including gas sensors (such as ammonia sensors) [19]. Silver nanowire, due to its high surface-to-volume ratio, good conductivity and a fast, simple, and cheap synthesis method has received great attention in different applications, such as sensing devices. In this study, the enhancement of the sensitivity of SAW sensors that are coated with silver nanowires with a poly(vinylpyrrolidone) (PVP) capping layer was investigated.

## 2. Materials and Methods

### 2.1. Chemicals

Poly(vinylpyrrolidone) (MW ~55,000), silver nitrate (AgNO_3_), and isopropyl (99.5%) were purchased from Sigma-Aldrich (St. Louis, MO, USA), Ethylene glycol (EG), sodium chloride (NaCl 99%), acetone, and ethanol were purchased from Merck Co., Kenilworth, NJ, USA.

### 2.2. Instruments and Devices

The following instruments and devices were used: a hotplate and stirrer, a syringe and syringe pump (KD Scientific, Holliston, MA, USA), a centrifuge device (Hettich, Buckinghamshire, UK), a sonication device, an electrospray device (which consists of two parts—a sensor holder, which rotates with a specific rate and is exposed to a negative charge, and a second part, an injector to spray the material with a positive charge), a SAW device (SAW Components GmbH, Dresden, Germany), an oscilloscope (Tektronix, Beaverton, OR, USA) to measure the frequency shift before and after coating, a sensor measurement setup (which includes 7 SAW devices, one of which is used as a reference that is closed with a lid to block the influence of the gases, and the other six of which are exposed to humidity at the same time), an optic microscope (Carl Zeiss, Oberkochen Germany), and a Scanning Electron Microscope (SEM) with a maximum resolution of 2 nm (JEOL JSM.6335F, Peabody, MA, USA) .

### 2.3. Synthesis of Silver Nanowires

First, 10 mL of a 0.45 M EG (ethylene glycol) solution of PVP was prepared, and 7 mg of NaCl was added afterward. The solution was poured into a two-necked round flask to be stirred and heated at 170 °C. A second solution was prepared, and 0.12 M AgNO_3_ was added to 5 mL of EG. This was injected dropwise into the PVP solution by an injection pump at a rate of 5 mL/h. The mixture was stirred and heated at the same temperature for an additional 30 min after injection. The solution color changes to a yellowish-brown. The final solution was cooled to room temperature, while it was stirring. After keeping the solution in a stable state for 2 days, the supernatant was decanted and the solid layer was washed and centrifuged 5 times with acetone and 5 times with ethanol [20]. After all purification processes, the final precipitate, silver nanowires (AgNWs) with a capping layer of PVP, was dispersed in isopropyl. Before dispersion in isopropyl, the weight of the solid precipitate was measured with a precision digital scale in order to reach a specific concentration of the solution.

### 2.4. SAW Device Preparation

A 433 MHz frequency dual-port resonator Rayleigh-SAW device (SAW Components GmbH, Dresden, Germany) mounted on a TO-39 socket was employed. This was washed via dipping in acetone and ethanol, each for 5 min. The reference frequency was measured with an oscilloscope to compare with its frequency after coating.

### 2.5. Electro-Spray Coating

**Figure 1:**
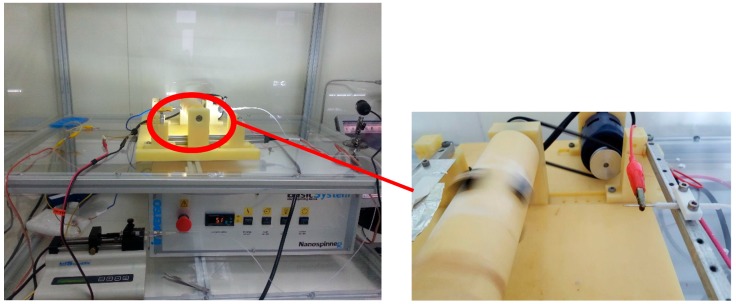
Photograph of the electrospraying system.

**Figure 2:**
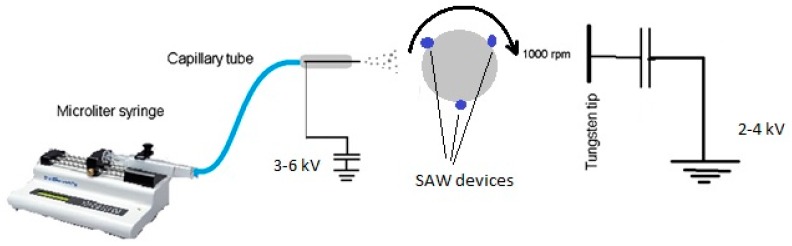
Schematic diagram of the electrospraying system.

The electro spraying system consists of a two-compartment setup with a sample holder in which sensors are placed on it and spins at 1000 rpm, exposing the sensors to a negative discharge cloud and the positive electrospray mist. A schematic diagram and its photograph are given in Figure 1 and Figure 2.

A syringe was filled with the solutions and placed in the syringe pump. The solution was injected at a constant rate into the sensors that were mounted on the rotator. As mentioned above, in order to obtain sensors with different sensitivities, we changed the amount of coating material on the SAW device. To that end, seven different SAW sensors were created by changing the injection rate, the volume, and the concentration of the injected solution. The details for these seven sensors are given in Table 1.

## 3. Results and Discussion

### 3.1. Characterization of Synthesized Silver Nanowires

**Figure 3:**
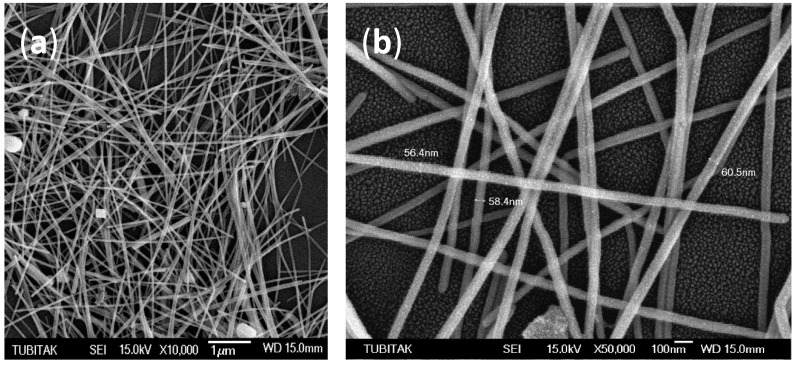
SEM images of silver nanowires (AgNWs): (**a**) the existing nanoparticles among the nanowires; (**b**) the dimensions of the nanowires.

The morphology and size of the polyol-synthesized silver nanowires were investigated via SEM analysis, and the results are shown in Figure 3.

The few silver nanoparticles that can be seen in Figure 3a are normal and inevitable in polyol synthesis. These nanoparticles have no significant effect on our experiments and results. The nanowires have an average length of 15 µm and an average width of 60 nm (Figure 3b). All wires are covered with a PVP capping agent, and no agglomeration is evident in the SEM photos.

**Figure 4:**
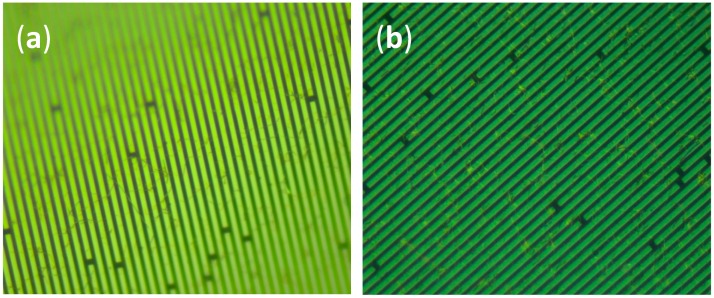
Optic microscope photos of IDTs coated with AgNWs by the electrospray coating method. (**a**) Sensor S6; (**b**) Sensor S7.

To observe the morphology of AgNWs on the surface of the SAW device and the inter-digital transducers (IDTs), we used an optic microscope with high magnification power and resolution. Figure 4 shows two different AgNW-coated IDT transducers (Sensors S6 and S7). Both photos show few silver nanowires on top of the fingers and between them. There is no connection between most of them, which demonstrates the very low electrical connectivity of the film. Although the amount of material sprayed on the surface of Sensor S7 (Figure 4b) is different in comparison to Sensor S6 (Figure 4a), the difference is not distinguishable on the images. The reason lies in the weight difference at the microgram or nanogram scales.

### 3.2. Characterization of the AgNW-Coated SAW Sensor

As was explained, the conductivity and mass of the sensing film are the parameters that change the frequency shift of the SAW device. Thus, we prepared SAW sensors with different coating loads of silver nanowires to investigate the humidity sensor response and SAW device characterization dependency on the mass and conductivity of the sensing film. To that end, seven different SAW sensors were made by changing the injection rate, the volume, and the concentration of the injected solution. The details for these sensors are given in Table 1.

In the primary investigation, it was concluded that no frequency shift was obtained from SAW devices with a high load of AgNWs, which is conductive. A conductive SAW device, which was obtained in the experiments, resulted from spraying 3 mL of the AgNW solution with a concentration of 0.5 mg/mL at an injection rate of 2 mL/h. This was also observed in the work of Cihat Tasaltin et al. [6], as they state that the active range of the acoustoelectric effect of the SAWs (the highest change of wave velocity) was found to be in the range of $0.4<\frac{\sigma_{s}}{V_{0}C_{s}}<1.7$. This range also includes the maximum point of insertion loss (IL). $\sigma_{s}=\sigma h$ is the surface conductivity, and $C_{s}=\varepsilon_{p}+\varepsilon_{0}$ is the concentration of the solution of the sensitive material (the total dielectric potential associated with the SAW and the carriers of the electric charge in the gas-sensitive film leads to a decrease in the velocity). V_0_ is the sound velocity in the crystal without any sensitive coated materials.

The desired frequency shift was obtained from other sensors with coat that was less thick. However, they were not conductive. The comparison of the measured frequencies with the duration of electro-spraying and the concentration of the sprayed solution showed a direct correlation.

### 3.3. Sensor Measurement Results

**Figure 5:**
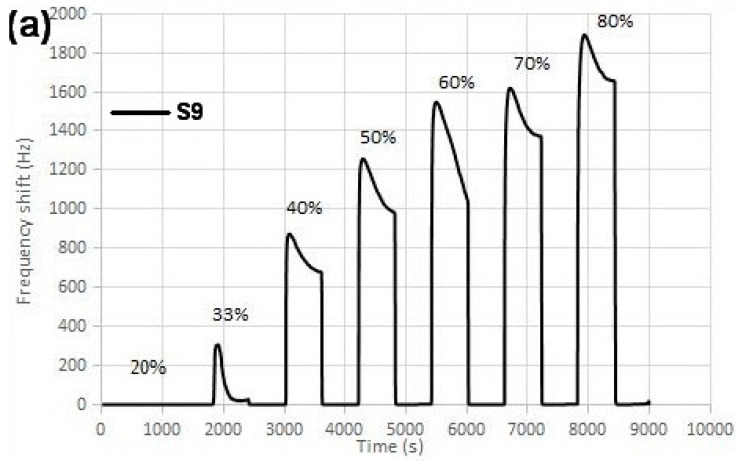

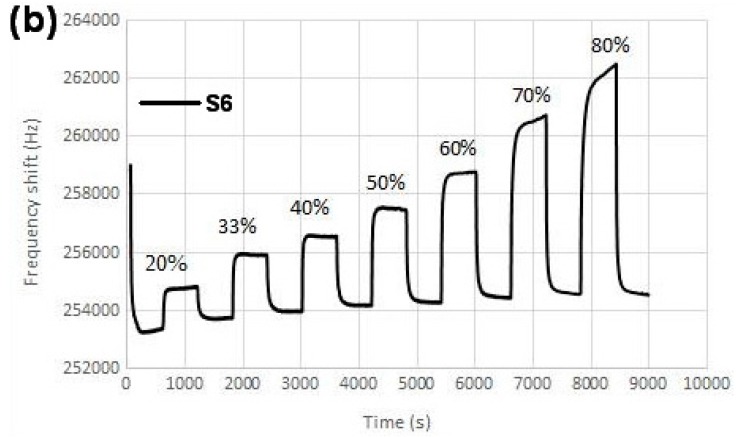
(**a**) An uncoated SAW sensor (S9) and (**b**) an AgNW-coated SAW sensor (S6) response against humidity in seven different humidity percentage in the range of 20%–80% RH. The baseline corresponds to 0% RH.

Sensor measurements were performed against humidity. The measurements were done twice for each of the sensors. The concentration humidity varied in a range from 20% RH to 80% RH. One uncoated sensor was tested as well to compare the results of the coated sensors against it and to check the sensitivity improvement. The acquired data was gathered in a spread sheet in order to draw the frequency graph versus the time for each sensor (Figure 5). The graph for Sensor S6, which was coated with a higher volume and concentration of AgNW solution, is shown in Figure 5b. The graph shows frequency shift responses to the changes in humidity percentage. 

**Figure 6:**
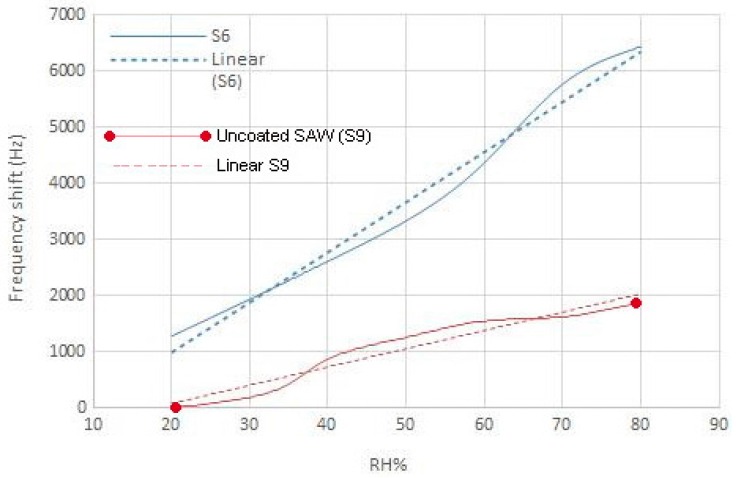
Sensitivity graphs of Sensor S6 and the uncoated SAW device.

Frequency vs. RH% graph, which indicates sensor sensitivity, for Sensor S6 and the uncoated one, is shown in Figure 6. It shows that the sensitivity of the SAW sensor with a AgNW-coated surface is higher than the uncoated SAW sensor. For Sensor S6, the frequency shift in 80% RH is about 6.5 kHz, which is three times greater than the uncoated sensor at 80% RH.

**Figure 7:**
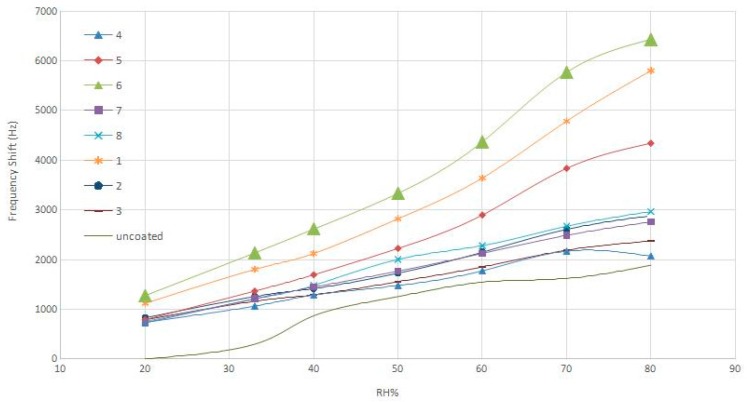
Sensitivity of all AgNW-based SAW sensors against humidity.

The sensitivity graph for all seven sensors is shown in Figure 7. Considering the data in Table 1 and Figure 7, it can be concluded that the sensitivity increases as the amount of coated AgNWs on the sensor grows. 

## 4. Conclusions

The achieved results from the research illustrate that SAW sensors work perfectly against humidity, and its response can be enhanced by coating a hydrophilic material on its surface as a sensing layer. AgNWs covered with PVP, because of its high surface-to-volume ratio and high hydrophilic property, are a good candidate for this purpose. The electrospray coating method was helpful in obtaining ideal samples with various amounts of nanomaterials on the surface. Sensor measurement results demonstrated that the sensitivity increases as the thickness of coated AgNWs on the sensor increases. It is also concluded that mass loading is a dominant factor in AgNW-based SAW humidity sensors. 

## Figures and Tables

**Table 1:** Parameters of AgNW-coated SAW sensors by the electrospray method.

No	Rotator	Volume	Injection Rate	Concentration	Frequency Shift in Air
S1	1000 rpm	0.75 mL	2 mL/h	0.25 mg/mL	240 KHz
S2	1000 rpm	0.25 mL	2 mL/h	0.5 mg/mL	165 KHz
S3	1000 rpm	0.25 mL	2 mL/h	0.25 mg/mL	123 KHz
S4	1000 rpm	0.25 mL	1 mL/h	0.25 mg/mL	91 KHz
S5	1000 rpm	0.25 mL	1 mL/h	0.75 mg/mL	220 KHz
S6	1000 rpm	0.5 mL	1 mL/h	0.5 mg/mL	262 KHz
S7	1000 rpm	0.25 mL	1 mL/h	0.5 mg/mL	158 KHz
S8	1000 rpm	0.5 mL	1 mL/h	0.25 mg/mL	188 KHz
S9	-	-	-	Uncoated SAW Device	-

## References

[1] Yamazoe N., Shimizu Y. (1986). Humidity sensors: Principles and applications. Sens. Actuators.

[2] Chen Z., Lu C. (2005). Humidity sensors: A review of materials and mechanisms. Sens. Lett..

[3] Kolpakov S.A., Gordon N.T., Mou C., Zhou K. (2014). Toward a new generation of photonic humidity sensors. Sensors.

[4] Consales M., Buosciolo A., Cutolo A., Breglio G., Irace A., Buontempo S., Petagna P., Giordano M., Cusano A. (2011). Fiber optic humidity sensors for high-energy physics applications at CERN. Sens. Actuators B Chem..

[5] Lin C.-M., Chen Y.-Y., Felmetsger V.V., Senesky D.G., Pisano A.P. (2012). AlN/3C–SiC Composite Plate Enabling High-Frequency and High-Q Micromechanical Resonators. Adv. Mater..

[6] Blank T.A., Eksperiandova L.P., Belikov K.N. (2016). Recent trends of ceramic humidity sensors development: A review. Sens. Actuators B Chem..

[7] Tasaltin C., Ebeoglu M.A., Ozturk Z.Z. (2012). Acoustoelectric effect on the responses of saw sensors coated with electrospun ZnO nanostructured thin film. Sensors.

[8] Hong H.-S., Chung G.-S. (2014). Controllable growth of oriented ZnO nanorods using Ga-doped seed layers and surface acoustic wave humidity sensor. Sens. Actuators B Chem..

[9] Hong H.-S., Phan D.-T., Chung G.-S. (2012). High-sensitivity humidity sensors with ZnO nanorods based two-port surface acoustic wave delay line. Sens. Actuators B Chem..

[10] Guo Y.J., Zhang J., Zhao C., Ma J.Y., Pang H.F., Hu P.A., Placido F., Gibson D., Zu X.T., Zu H.Y. (2013). Characterization and humidity sensing of ZnO/42° YX LiTaO_3_ love wave devices with ZnO nanorods. Mater. Res. Bull..

[11] Penza M., Antolini F., Vittori-Antisari M. (2005). Carbon nanotubes-based surface acoustic waves oscillating sensor for vapour detection. Thin Solid Films.

[12] Buvailo A., Xing Y., Hines J., Borguet E. (2011). Thin polymer film based rapid surface acoustic wave humidity sensors. Sens. Actuators B Chem..

[13] Ricco A.J., Martin S.J., Zipperian T.E. (1985). Surface acoustic wave gas sensor based on film conductivity changes. Sens. Actuators.

[14] Penza M., Aversa P., Cassano G., Wlodarski W., Kalantar-Zadeh K. (2007). Layered saw gas sensor with single-walled carbon nanotube-based nanocomposite coating. Sens. Actuators B Chem..

[15] Tashtoush N.M. (1996). SAW Humidity Sensor and an Environmental Electronic Nose System. Ph.D. Thesis.

[16] Li D.J., Zhao C., Fu Y.Q., Luo J.K. (2014). Engineering silver nanostructures for surface acoustic wave humidity sensors sensitivity enhancement. J. Electrochem. Soc..

[17] Li Y., Li P., Yang M., Lei S., Chen Y., Guo X. (2010). A surface acoustic wave humidity sensor based on electrosprayed silicon-containing polyelectrolyte. Sens. Actuators B Chem..

[18] Sheng L., Chen D., Chen Y. (2011). A surface acoustic wave humidity sensor with high sensitivity based on electrospun MWCNT/Nafion nanofiber films. Nanotechnology.

[19] Dubas S.T., Vimolvan P. (2008). Humic acid assisted synthesis of silver nanoparticles and its application to herbicide detection. Mater. Lett..

[20] Coskun S., Aksoy B., Unalan H.E. (2011). Polyol synthesis of silver nanowires: An extensive parametric study. Cryst. Growth Des..

